# How Cells Cope with Obesity

**DOI:** 10.1371/journal.pbio.1001077

**Published:** 2011-06-07

**Authors:** Caitlin Sedwick

**Affiliations:** Freelance Science Writer, San Diego, California, United States of America

**Figure pbio-1001077-g001:**
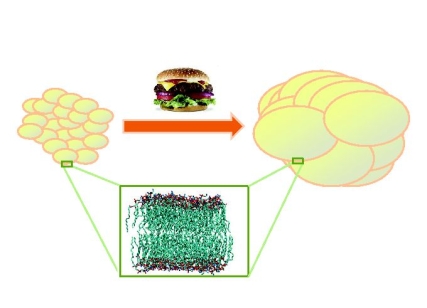
Remodeling of adipose tissue membrane lipids as adaptation to acquired obesity: benefits and costs.

[Fig pbio-1001077-g001]Currently, more than 2.7 million American adults are diagnosed as obese each year. Many of these people suffer from a disorder known as metabolic syndrome, which includes symptoms like hypertension and elevated blood cholesterol. They are also at risk for developing additional diseases such as heart disease and diabetes mellitus. In fact, obesity may be a major cause of all these problems … the question is, why? That's the question Kirsi H. Pietiläinen, Antonio Vidal-Puig, Matej Orešič, and colleagues set out to address in their paper published in this month's *PLoS Biology*.

One prominent theory explaining how obesity could drive advanced disease states is the “adipose tissue expandability” hypothesis. This theory is based on the idea that as excess fat is added to the body, its fat compartment (adipose tissue) expands as more fat-storing cells are made and existing ones increase in size. However, expandability of the fat compartment is finite; like a water balloon, the fat compartment can only expand so much before leaks develop. For the body, “leaks” in the fat compartment manifest as depositions of fat in tissues where its presence can be disruptive to normal function—as happens in morbidly obese individuals. But, lower levels of expansion are tolerated—possibly due to compensatory mechanisms—so that tissues and systems can function normally. So what is going on in the body when the fat compartments are expanded but not yet full?

To explore this question in humans, the authors decided to compare adipose tissue and health parameters amongst several sets of monozygotic twins. In each twin pair, one twin was obese (but still healthy, i.e., not morbidly obese), while the other twin exhibited a normal body mass index. Because monozygotic twins share the same DNA and early upbringing, the impact of these factors on adult body mass phenotypes is controlled for, leaving other factors such as adult diet and lifestyle choices as the major remaining variables.

When the authors compared dietary intake within twin sets, they found that obese twins had lower amounts of polyunsaturated fatty acids in their diets than did their non-obese counterparts. The kinds of fats a person eats can affect what types of lipids are present in the body, so the authors next compared the lipid content of obese versus non-obese twins' adipose tissue. In fact, they found the obese people had higher amounts of certain types of phospholipids in their adipose tissues than did their non-obese co-twins.

This finding is interesting because cell membranes are primarily composed of lipids, and different lipids can alter membranes' physical properties. For example, lipids with larger head groups, or longer or branched side chains are found to pack less tightly in membranes, making the membranes that host them more fluid. When the authors used computers to model the effect these different lipids have on adipose cell membranes, they found that the new lipids observed in obese twins' adipose cells balance each other in such a way that membrane fluidity overall is unaffected. The authors concluded that lipid content changes in obese individuals might actually be an adaptation that serves to preserve membrane function as the cells expand. However, additional analyses suggested that this adaptation can only go so far, and breaks down in the morbidly obese.

To find out how this adaptive response is achieved, the authors conducted a statistical network analysis (based on clinical and genetic variables) to try to identify the regulatory mechanisms that underpin these changes. This analysis showed that the gene encoding the fatty acid elongase Elovl6 might be involved in fatty acid remodeling in obese people. Indeed, when the researchers reduced Elovl6 expression in an adipocyte cell line, they found the cells could no longer maintain the right level of the adaptive lipids observed in obese twins.

Collectively, the authors' data point to some of the mechanisms the body may use to adapt to excess fat. These results may also help explain why obese people are at risk for developing inflammatory disorders like diabetes mellitus: the kinds of lipids that accumulate in obese people's adipocytes are precursors for compounds that are known to aggravate the immune system. Of course, these findings need to be validated by more studies, including ones using transgenic animals. Nonetheless, they represent a valuable angle from which to approach the problem.

**Pietiläinen KH, Róg T, Seppänen-Laakso T, Virtue S, Gopalacharyulu P, et al. (2011) Association of Lipidome Remodeling in the Adipocyte Membrane with Acquired Obesity in Humans. doi:10.1371/journal.pbio.1000623**

